# Altered activation and connectivity in a hippocampal–basal ganglia–midbrain circuit during salience processing in subjects at ultra high risk for psychosis

**DOI:** 10.1038/tp.2017.174

**Published:** 2017-10-03

**Authors:** T Winton-Brown, A Schmidt, J P Roiser, O D Howes, A Egerton, P Fusar-Poli, N Bunzeck, A A Grace, E Duzel, S Kapur, P McGuire

**Affiliations:** 1 Department of Psychosis Studies, Institute of Psychiatry, Psychology and Neuroscience King’s College London, London, UK; 2 Monash Alfred Psychiatry Research Centre, Monash University, Melbourne, VIC, Australia; 3 Institute of Cognitive Neuroscience, University College London, London, UK; 4 Psychiatric Imaging, MRC Clinical Sciences Centre, Hammersmith Hospital, London, UK; 5 Early Psychosis: Intervention and Clinical-prediction (EPIC) Lab, Department of Psychosis Studies, Institute of Psychology, Psychiatry and Neuroscience, King's College London, London, UK; 6 OASIS Service, South London and Maudsley (SLaM) NHS Foundation Trusts, Beckenham, UK; 7 Institute of Psychology, University of Luebeck, Luebeck, Germany; 8 Department of Systems Neuroscience, University Medical Center Hamburg-Eppendorf, Hamburg, Germany; 9 Department of Neuroscience, University of Pittsburgh, Pittsburgh, PA, USA; 10 Faculty of Medicine, Dentistry and Health Sciences, University of Melbourne, Melbourne, VIC, Australia

## Abstract

Animal models of psychosis propose that abnormal hippocampal activity drives increased subcortical dopamine function, which is thought to contribute to aberrant salience processing and psychotic symptoms. These effects appear to be mediated through connections between the hippocampus, ventral striatum/pallidum and the midbrain. The aim of the present study was to examine the activity and connectivity in this pathway in people at ultra high risk (UHR) for psychosis. Functional magnetic resonance imaging was used to compare neural responses in a hippocampal–basal ganglia–midbrain network during reward, novelty and aversion processing between 29 UHR subjects and 32 healthy controls. We then investigated whether effective connectivity within this network is perturbed in UHR subjects, using dynamic causal modelling (DCM). Finally, we examined the relationship between alterations in activation and connectivity in the UHR subjects and the severity of their psychotic symptoms. During reward anticipation, UHR subjects showed greater activation than controls in the ventral pallidum bilaterally. There were no differences in activation during novelty or aversion processing. DCM revealed that reward-induced modulation of connectivity from the ventral striatum/pallidum to the midbrain was greater in UHR subjects than controls, and that in UHR subjects, the strength of connectivity in this pathway was correlated with the severity of their abnormal beliefs. In conclusion, ventral striatal/pallidal function is altered in people at UHR for psychosis and this is related to the level of their psychotic symptoms.

## Introduction

Two neural hallmarks of psychosis are increased presynaptic striatal dopamine function^1^ and structural and functional abnormalities in the hippocampus.^2^ Both elevated striatal dopamine function^3, 4^ and hippocampal abnormalities^5, 6^ are also evident in subjects at ultra high risk (UHR) for psychosis, suggesting that they are associated with an increased vulnerability for psychosis. Animal models of psychosis propose that these two abnormalities may be linked via a polysynaptic pathway involving the hippocampus, basal ganglia and midbrain.^7, 8^ Cognitive models of psychosis propose that psychotic symptoms develop as a result of altered salience processing.^9^ Taken together, these models propose that the presence of a salient stimulus in healthy subjects is associated with increased hippocampal activity and descending glutamatergic drive to GABAergic neurons in the ventral striatum. This suppresses the activity of GABAergic neurons in the ventral pallidum that normally inhibit the activity of dopaminergic neurones in the midbrain.^10, 11^ Dopamine function is thus enhanced by salient stimuli and this hippocampal–basal ganglia–midbrain loop is thought to mediate the attribution of salience to environmental signals based on the context.^12^ In patients with psychosis, it is suggested that resting overactivity in the ventral hippocampus drives the ventral striatum to potently inhibit the ventral pallidum, markedly increasing the number of spontaneously active midbrain dopamine neurons,^7, 13^ and leading to an increase in dopaminergic activity that is uncoupled from context. Data from multimodal neuroimaging studies in UHR individuals are consistent with this model, indicating that the relationship between hippocampal activity and glutamate levels with striatal dopamine function is significantly altered compared to that in controls.^14^

The aberrant salience hypothesis of psychosis^9, 15, 16^ proposes that chaotic dopamine release can perturb salience processing in two ways. If phasic dopamine release is dysregulated and coincides with the processing of stimuli that would normally be irrelevant, these may become inappropriately salient. Conversely, if the phasic dopamine release that normally occurs in response to contextually relevant cues is impaired, stimuli that would normally be salient may become less so. This is consistent with data from recent studies in UHR subjects and in patients with psychosis, which report impairments in both forms of salience processing.^17, 18, 19^

Most previous functional magnetic resonance imaging (fMRI) studies in psychosis have examined the attribution of salience of stimuli in relation to reward.^20, 21^ However, in real-life stimuli may also be salient for other reasons. Analogous to a visual ‘saliency map’,^22^ higher order elements such as reward, novelty and aversion may interact to help determine the most salient stimuli for the organism in its current state and context. Whether the putative alterations in salience processing in psychosis are specific to reward or also apply to other dimensions is unspecified by the model^9^and remains to be established.

The first aim of the present study was to use fMRI to assess activation in a hippocampal–basal ganglia–midbrain circuit during reward, novelty and aversion processing in subjects at UHR for psychosis. We then sought to investigate whether effective connectivity within this network is perturbed in UHR subjects, using dynamic causal modelling (DCM).^23^ Finally, we explored the relationship between the altered activation and connectivity strengths in UHR subjects and the severity of their psychotic symptoms. Our first prediction was that activation within the putative hippocampal–basal ganglia–midbrain circuit would be significantly altered in UHR subjects compared to controls. We expected these differences to be evident when salience was related to novelty and aversion, as well as to reward processing. Our second prediction was that connectivity within this network in UHR subjects would also be altered. Finally, we predicted that altered activation and connectivity in UHR subjects would be related to the severity of psychotic symptoms.

## Materials and methods

### Subjects

Twenty-nine individuals who met Comprehensive Assessment of At-Risk Mental States (CAARMS)^24^ criteria for the UHR state were recruited from Outreach and Support in South London (OASIS), a clinical service for people at high risk for psychosis.^25^ Inclusion criteria required the presence of one or more of the following: (i) attenuated psychotic symptoms (APS), (ii) frank psychotic symptoms of <1 week’s duration (Brief Limited Intermittent Psychotic symptoms) or (iii) schizotypal personality disorder or a first-degree relative with psychosis, plus a marked recent decline in psychosocial functioning (genetically determined risk).^24^

Thirty-two healthy controls (HCs) were recruited by advertisement from the same geographical area. Absence of psychiatric illness history was confirmed with the Mini International Neuropsychiatric Inventory.^26^ None of the control subjects had a history of neurological illness, DSM-IV drug or alcohol dependence, though a significant minority in both groups were smokers and admitted previous illicit substance use (Table 1). All UHR subjects were antipsychotic medication naive, while two UHR subjects were prescribed SSRI medication at the time of scanning (Citalopram 20 mg). All control subjects were medication naive. HC subjects were marginally older and had more education than UHR subjects—age matching of HC subjects was also to a third older group of first episode psychosis subjects not included in the current study. All subjects provided informed written consent to participate and the study was approved by the South London and Maudsley Research Ethics Committee. See Table 1 for demographic and clinical characteristics of the study sample.

### The Salience Integration Task

The Salience Integration Task (SIT) is a modification of the Monetary Incentive Delay task.^27^ It was designed to permit the simultaneous study of three salience dimensions, reward, novelty and aversion. All three dimensions were inherent to the picture cue: an indoor–outdoor setting indicated whether the trial held the chance of a 20-pence reward (reward-predicting cues, 80% of indoor scenes), or had no incentive relevance (neutral cues, 80% of outdoor scenes). Half of the cues were indoor scenes and half were outdoor. To generate the novelty dimension, half of both indoor and outdoor scenes were familiarized beforehand (displayed for 500 ms three times immediately before scanning) leaving half as novel images. Finally half of all of the pictures (indoor and outdoor, familiar and novel) were aversive pictures, taken from the International Affective Picture System (IAPS),^28^ with a minimum arousal level of 3.0 (mean (s.d.) arousal=5.6 (0.86)). There were no significant differences in arousal levels across reward and novelty dimensions. This resulted in a 2x2x2 factorial event-related design, in which the orthogonal cue variables reward prediction, novelty and aversion were manipulated separately, yielding eight experimental conditions that were used to form contrasts in the subsequent behavioural and fMRI analyses of main effects and interactions. As the task was designed to generate comparable probes of each of the three dimensions of salience, reward delivery was not contingent upon speed of response—this was in order to reduce attentional bias towards the reward aspect of the task relative to the aversion and novelty dimensions.

Following presentation of the picture cue for 1500 ms, subjects were instructed to press a button with their dominant hand index finger as fast as possible, regardless of cue type, aside from two pre-assigned and randomly inserted ‘No-Go’ pictures (one indoor, one outdoor). These pictures served as attentional controls and encouraged processing of scene detail. After the picture cue, a black fixation cross on a grey background followed for 1000–2500 ms, followed in rewarded trials by picture of a 20-p coin with ‘WIN!’ in green text underneath, or in unrewarded trials a similar shaped blank icon with the words ‘No Money Available’ in red text for 750 ms, followed by a further fixation cross for 150–1650 ms (Supplementary Figure 1). Subjects were told to respond quickly and accurately for each trial, although reward contingencies were predetermined for each trial to provide a fixed reinforcement ratio (0.8). There were 35 trials in each of the eight response categories and 35 No-Go trials giving a total of 315 trials. Each trial lasted 4.9 s giving a total paradigm length of 25 min 43.5 s. At 1 and 24 h following the online task subjects rated recognition and emotional distress from each previously shown picture intermixed with new distractor pictures (Supplementary Methods, Supplementary Figure 2). This was to ensure the validity of probes in terms of memory encoding and motivational effects.

### Behavioural group comparison

Behavioural comparisons were made on reaction time and recognition accuracy. For group comparisons of reaction times, we conducted a repeated measures analysis of variance with reward, novelty and emotion as within-subject variables, and group entered as a between-subject variable. Similar analyses of recognition rates utilized measures of both hit rate and discrimination accuracy (hit rate corrected for false alarms, see Supplementary Methods), with repeated measures analyses of variance with reward, novelty, emotion and recall session as within-subject variables and group as between-subject variable.

### fMRI analysis

Detailed descriptions of the fMRI data acquisition and pre-processing are provided in the Supplementary Methods. For between-group comparisons, a two-sample *t*-test was performed at the second (group) level for each of the main effects of interest, reward, aversion and novelty, while covarying for age. As education may be expected to differ between groups, this was not included as a covariate in the main analysis. We performed whole-brain analyses at an uncorrected threshold of *P*<0.005 and applied small volume correction at a threshold of family-wise error (FWE) <0.05 within three pre-specified bilateral regions of interest: the midbrain, hippocampus/parahippocampal gyrus and ventral striatum/pallidum. Bonferroni correction was then applied for these three regions of interest (ROIs). For the hippocampus/parahippocampal regions of interest, we used anatomical masks from the automated anatomical labelling toolbox implemented in SPM 8^[ref. 29]^ (Supplementary Figure 3A). For the midbrain ROI, we visualized the substantia nigra/ventral tegmental area as bilateral dark stripes in midbrain slices on the acquired mp2rage T1 sequence and created a study-specific mask based on the landmarks in Bunzeck and Duzel^30^ using Mricron software (http://www.mccauslandcenter.sc.edu/mricro/mricron/) (Supplementary Figure 3B). The ventral striatum/pallidum ROI was similarly created and comprised the ventral anterior portion of the head and body of caudate, nucleus accumbens, ventral putamen and pallidum (Supplementary Figure 3C).

### Dynamic causal modelling

We used DCM12 with SPM12 (v6225) to compute effective connectivity within the hippocampal–basal ganglia–midbrain loop. In DCM for fMRI, the dynamics of the neural states underlying regional BOLD responses are modelled by a bilinear differential equation that describes how the neural states change as a function of endogenous interregional connections, modulatory effects on these connections and driving inputs.^23^ The endogenous connections represent constant coupling strengths, whereas the modulatory effects represent context-specific and additive changes in coupling (task-induced alterations in connectivity). The modelled neuronal dynamic is then mapped to the measured BOLD signal using a hemodynamic forward model.^31^ In the current study, based on the group-level fMRI findings, we explicitly explored how the coupling strengths between hippocampus, ventral striatum/pallidum and midbrain were changed by reward-predicting cues (modulatory effect).

### Volumes of interest definition and time series extraction

Based on the circuit proposed by the animal model^7, 10, 32^ and our group level fMRI findings of altered left ventral pallidum and midbrain activation, we selected a left hemispheric network. We used the same hippocampus and ventral striatum/pallidum masks as used for the fMRI analysis. In contrast to the fMRI analysis, we selected the whole midbrain instead of the substantia nigra/ventral tegmental area to extract more voxels for the DCM analysis. The whole ventral striatal/pallidal ROI was utilized for the same reason. Subject-specific time series were extracted from the anatomically defined masks using the *t* contrast rewarding-predicting versus non-reward-predicting cues at *P*=0.1 (adjusted for effect of interest *F* contrast). One UHR subject showed no activated voxels at this threshold and was therefore excluded from the DCM analysis.

### Model space construction

All stimuli (reward-predicting and non-reward-predicting cues) were used as driving input into our models. All three regions were selected as putative input regions: the hippocampus receives visual input via the visual–perirhinal–hippocampal stream,^33, 34^ the striatum from visual–corticostriatal pathways^35, 36^ and the midbrain through the retinotectal and tectonigral pathway.^37, 38^ We created six different potential model variations depending on where reward-predicting cues might modulate hippocampal–ventral striatum/pallidum–midbrain connections. For a graphic summary of the model space see Supplementary Figure 4.

### Bayesian model selection and averaging

We used Bayesian model selection (see Supplementary Methods for more information) to determine the most plausible model and family of models (F1: bilinear versus F2: non-linear) for both groups separately. To compare DCM parameters across groups, Bayesian model averaging was used to average the posterior parameter estimates over each model from the winning family for each group separately, weighted by the posterior model probabilities.^31^ Thus, models with a low posterior probability contribute little to the estimation of the marginal posterior. We used a univariate analysis of variance with age as covariate to compare these connectivity parameters between UHR subjects and HCs (Bonferroni-corrected for multiple connections).

### Relation between activation and connectivity and APS

Using Pearson correlation analysis, we tested the relationship between the significantly altered activation and connectivity strengths between groups and the formation of APS in UHR subjects as measured by CAARMS scores. Summary scores were calculated for each of the three positive symptoms subscales (abnormal though content, perceptual abnormalities and speech abnormalities) by multiplying severity and frequency scores. Influential outliers were detected using a critical value of Cook’s *D* >4/*n*−*k*−1, for *k* predictors and *n* cases.^39^

## Results

### Demographics

Demographics and clinical details of the sample are provided in Table 1. Function was lower in UHR subjects, who were also slightly younger and less educated than controls.

### Behavioural data

#### Reaction time

Across the whole study sample there were main effects of novelty (*F*=15.771, *P*<0.001), and aversion (*F*=9.494, *P*=0.003) in slowing reaction times (Supplementary Figure 5A). There were significant interactions between reward and aversion (*F*=39.413, *P*<0.001), and between novelty and aversion (*F*=8.852, *P*=0.004)—within non-aversive trials there was a significant effect of reward in speeding trials, and of novelty in slowing trials (Supplementary Figure 5B). There was no significant group difference in reaction time, and no significant group by task reaction time interactions.

#### Recognition

The mean(s.d.) hit rate was 55%(14%). Across the whole study sample there were main effects of reward (+7.7%, *F*=46.664, *P*<0.0001), novelty (−14.5%, *F*=172.416, *P*<0.0001), aversion (+14.6%, *F*=49.232, *P*<0.0001) and recall session (+9.1% *F*=62.185, *P*<0.0001) on recognition hit rate. Similar main effects were found on discrimination accuracy, with a reversal of the effect of reward on hit rate driven by a disproportionate effect of reward on raising the false alarm rate (Supplementary Figure 6).

There was a main effect of group on hit rate with UHR subjects showing lower hit rates (−10.5%, *F*= 8.412, *P*=0.005) but not lower discrimination accuracy (−4.9%, *F*=3.11, *P*=0.083). There was a significant group × aversion interaction on hit rate (*F*=4.493, *P*=0.038) but not discrimination accuracy (*F*=0.153, *P*=0.698) and a significant group × novelty interaction on both hit rate (*F*=5.209, *P*=0.026) and discrimination accuracy (*F*=5.954, *P*=0.018) (Supplementary Figure 6).

### fMRI data

#### Effects of task in HC and UHR subjects

The main effects of task are reported at *P*<0.005 uncorrected within ROIs and across the whole brain, in healthy controls and in UHR subjects.

**Figure 1:**
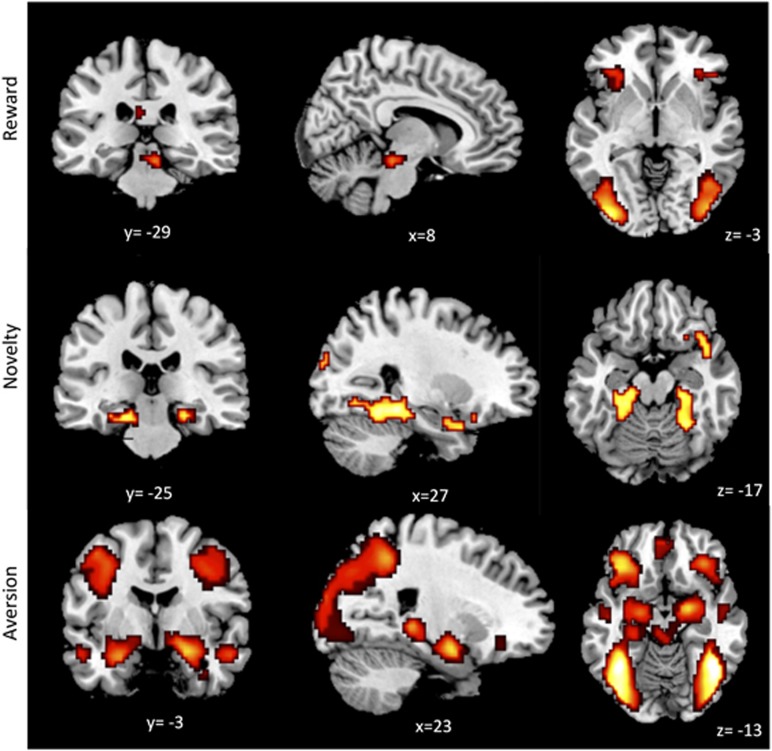
Salience Intigration Task effects in healthy control subjects. Reward predicting stimuli-related activation was seen in the midbrain, bilateral orbitofrontal cortices and visual areas. Novel stimuli-related activation was seen in the hippocampus and secondary visual cortices bilaterally. Aversive stimuli-related activation was seen in the amygdala, hippocampus and midbrain bilaterally, visual and fusiform cortices, and orbital, ventromedial and dorsolateral prefrontal cortices.

Reward (HC): within the ROI network, activation related to reward-predicting stimuli was evident in the midbrain (Supplementary Table 1A). Outside this network, there was activation in the orbitofrontal and primary and secondary visual cortices bilaterally (Figure 1a, Supplementary Table 1B).

Reward (UHR): within the ROI network, activation related to reward-predicting stimuli was evident in the ventral pallidum bilaterally, extending to left amygdala and right midbrain and bilateral hippocampi (Supplementary Table 2A). Outside this network, activation was evident in the primary and secondary visual cortices bilaterally, and in the left cingulate and inferior frontal gyri (Supplementary Table 2B).

Novelty (HC): within the ROI network, activation related to novel stimuli was evident in the hippocampal, parahippocampal and entorhinal cortices bilaterally. Outside this network, there was activation in the secondary visual cortices (Figure 1b, Supplementary Table 1).

Novelty (UHR): there was no activation related to novel stimuli within the ROI network. Outside this network, activation was evident in the left fusiform, cingulate and inferior occipital gyri, and the right inferior temporal and occipital gyri (Supplementary Table 2B).

Aversion (HC): within the ROI network, activation related to aversive emotional stimuli was found in the amygdala, hippocampus and midbrain bilaterally. Outside this network, there was activation in the primary/secondary visual and fusiform cortices, and the orbital, ventromedial and dorsolateral prefrontal cortices (Figure 1c, Supplementary Table 1A).

Aversion (UHR): within the ROI network activation related to aversive emotional stimuli was found in the amygdala and hippocampus bilaterally (Supplementary Table 2A). Outside this network, there was activation in the temporal, fusiform and inferior frontal gyri bilaterally (Supplementary Table 2B).

#### Group differences in brain activation

**Figure 2:**
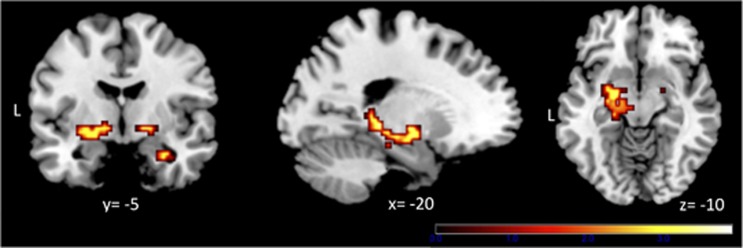
Ultra high-risk (UHR) subjects showed greater activation than healthy controls (HCs) in relation to reward-predicting stimuli in the ventral pallidum bilaterally and in the left midbrain/hippocampus. Images are displayed at a cluster-forming threshold of *P*<0.005.

Reward: UHR subjects showed significantly greater activation to reward-predicting cues than controls in the left ventral pallidum (pFWE=0.045, Figure 2, Table 2) and left midbrain (pFWE=0.039, Figure 2, Table 2). There were no areas where control subjects showed greater activation than UHR subjects.

Novelty: there were no significant group differences in activation related to novelty.

Aversion: there were no significant group differences in activation related to aversion.

#### Relationship between differential activation and APS

There were no significant correlations between group differences in activation related to reward processing and scores on the CAARMS positive symptom subscales.

### DCM results during reward anticipation

#### Bayesian model selection

The family of bilinear models was clearly superior than the family of non-linear models in both HC (excedance probability, EP(F1)=95%, EP(F2)=5%) and UHR subjects (EP(F1)=98%, EP(F2)=2%) (Supplementary Figure 6). Bayesian model selection comparison of single models revealed that model 1 was the best fitting model in both HCs (EP=81%) and UHR subjects (EP=65%) (Supplementary Figure 7).

#### Group differences in effective connectivity

**Figure 3:**
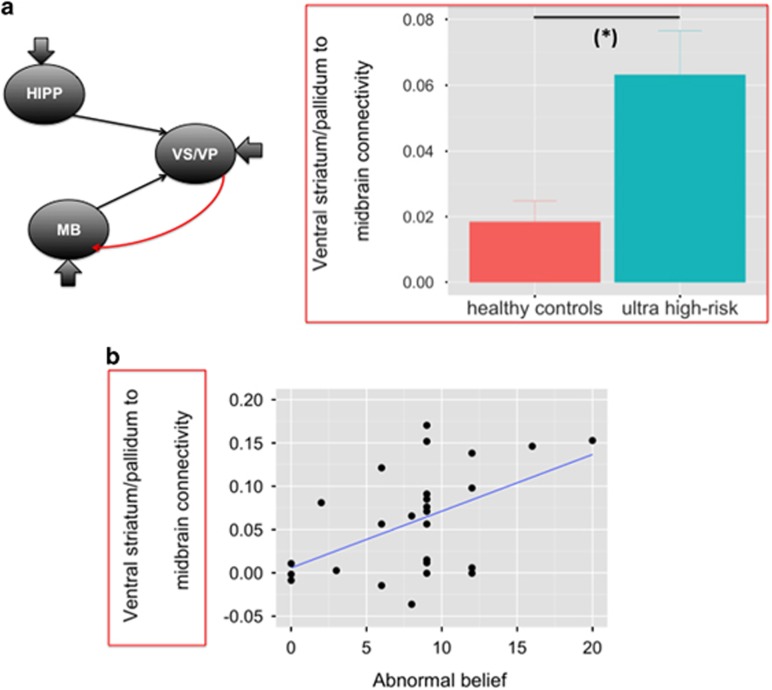
(**a**) Increased reward-induced modulation of ventral striatum/pallidum to midbrain connectivity in ultra high-risk individuals relative to healthy controls. (**b**) Positive correlation between reward-induced modulation of ventral striatum/pallidum to midbrain connectivity and abnormal beliefs (CAARMS ‘Unusual Thought Content’ item, severity × frequency) in ultra high risk subjects (*r*=0.499, *P*=0.009).

In our group-level analysis of effective connectivity, we were able to test for group differences in four parameters describing the reward-induced modulation of hippocampal–ventral striatum/pallidum–midbrain connections (Supplementary Table 3). We found that the reward-induced modulation of ventral striatum/pallidum to midbrain connectivity was significantly greater in UHR subjects than in HCs (*F*=10.729, *P*=0.002, Bonferroni corrected for multiple comparisons) (Figure 3a and Supplementary Table 3).

#### Relationship between ventral striatum/pallidum–midbrain connectivity and APS

In UHR subjects, there was a significant positive correlation between ventral striatum/pallidum to midbrain connectivity and abnormal beliefs (*r*=0.499, *P*=0.009; excluding two outliers, Figure 3b). There were no significant correlations between ventral striatum/pallidum to midbrain connectivity and the severity of other positive symptoms (perceptual abnormalities: *r*=0.226, *P*=0.268; disorganized speech: *r*=−0.120, *P*=0.558).

#### Analysis of UHR subtypes

All but one subject met criteria for UHR based on APS. This subject who met criteria based on genetically determined risk was removed from the main fMRI and connectivity analyses and the main results were not significantly altered.

#### Transition to psychosis

Four of twenty-nine UHR subjects transitioned to psychosis during the 2-year follow-up period (13%). There were no differences in baseline demographics, CAARMS scores or behavioural results between the HC, UHR transition (UHR-T) and UHR-non-transition (UHR-NT) groups. Similarly there were no significant differences in fMRI activation or connectivity measures.

## Discussion

To our knowledge, this is the first fMRI investigation of multidimensional salience processing in subjects at UHR subjects for psychosis. Although previous studies in psychosis have focused on reward-related salience,^21^ this work also investigated the roles of novelty and aversion. We also examined whether effective connectivity within the hippocampal–midbrain loop is altered during reward, aversion and novelty processing in UHR subjects, and whether alterations in activation and connectivity were related to the severity of APS.

### Activation during multidimensional salience processing

HC brain activation responses confirmed the validity of effects of each of the three probes of salience investigated. When processing salience related to reward anticipation, UHR subjects showed greater activation than controls in the ventral pallidum, a major projection target in the outflow path from the ventral striatum that provides resting inhibition of midbrain dopamine neurons.^8^ At a lower statistical threshold greater activation was also evident in the left hippocampal subiculum. These findings are consistent with our first hypothesis, and are in line with animal models,^8, 9, 13^ which indicate that altered ventral pallidal function is a key feature of aberrant salience processing in psychosis. Increased BOLD signal in this area is likely to largely reflect inputs,^40^ in this case, increased inhibitory afferents from ventral striatum, which thereby disinhibit midbrain dopamine neurons.^7^ The role of the ventral pallidum was also emphasized in a recent multivariate pattern analysis, which showed that ventral pallidum activation during reward anticipation contributed most to discriminating patients with schizophrenia from HCs.^41^

There have been five previous functional MRI studies of UHR subjects during reward anticipation. One reported ventral striatal hypoactivation,^42^ and four found no differences in activation between groups;^18, 43, 44, 45^ none report differences in the ventral pallidum. Besides differences in sample sizes and methodological approaches, these inconsistent findings may also reflect differences in the severity of symptoms in the UHR samples across studies. For example, a previous study in UHR subjects found that the magnitude of ventral striatal activation during reward anticipation was correlated with the severity of positive symptoms.^43^ They may also reflect developmental differences: a recent study of over 1500 adolescents found reward-related responses in the ventral striatum increased with increased polygenic psychosis risk scores.^46, 47^

Nevertheless, the finding of group differences in relation to reward processing is broadly consistent with most of the literature on salience in patients with established psychosis, which mainly comprises abnormalities of activation in the context of tasks involving reward. The most common finding in these studies has been of reduced ventral striatal activation.^20^ Our findings were in a nearby region, which may have an opposed function within the circuit of interest^12^ and they were of greater activation in UHR subjects than controls, which therefore may be consistent with these previous findings. They also raise an alternative possibility that the polarity of alterations in reward processing may vary with the stage of psychosis. This notion of a ‘hypersalient’ period prior to the onset of frank psychosis^9^ is supported by phenomenological accounts of the prodromal phase describing heightened vividness and increased salience and meaning from sensory stimuli.^48^ However, we did not detect significant behavioural differences between UHR subjects and controls groups—minor group differences in recognition performance related to novelty are difficult to interpret because of the effect of pre-familiarization. Although this might have been due to a lack of statistical power, previous studies using smaller samples have reported significant differences in performance of other salience tasks such as the Salience Attribution Task,^18^ suggesting that it may have been related to aspects of the salience integration task paradigm, such as celling effects and the lack of dynamically adjusted reward outcomes. These were necessary in the task design in order to enable testing reward, novelty and aversion concurrently without overtly biasing attention towards one aspect over the others.

In the present study, there were no significant group differences in activation during the processing of salience related to novelty. This is surprising, given that an altered sense of novelty has been described in the early stages of psychotic disorders^9, 48^ and there is evidence of disrupted novelty processing in patients with established psychosis.^49^ Similarly, there were no significant group differences in activation during the processing of salience related to aversion, which was unexpected in view of the prominence of emotional processing abnormalities in UHR subjects,^50, 51^ and the typically high levels of anxiety and depressive symptoms in this group.^52^ Although our findings might suggest that the processing of salience related to reward is more perturbed in UHR subjects than that related to novelty or aversion, the respective paradigms are unlikely to have placed identical demands on each type of salience processing, and it was not possible to reliably compare responses between salience types in this task.

### Hippocampal–basal ganglia–midbrain connectivity during reward anticipation

Although animal models of psychosis suggest that there is altered connectivity within a hippocampal–basal ganglia–midbrain circuit,^7, 8, 12^ there is limited evidence of this from neuroimaging studies in humans. We used a model-based approach to explore whether ventral pallidal hyperactivation was accompanied by altered functional coupling within the hippocampus–basal ganglia–midbrain circuit. We found that in UHR subjects, connectivity from the ventral striatum/pallidum to the midbrain during reward prediction was significantly greater than in controls. This is consistent with the notion that increased dopamine function in psychosis is driven by descending inputs from the hippocampus to the ventral striatum, which, via the ventral pallidum, projects to dopaminergic neurons in the midbrain.^7, 8, 12^ Moreover, we found that in UHR subjects, the greater the connectivity between ventral striatum/pallidum and midbrain during reward prediction, the greater the severity of abnormal beliefs. This represents some of the first human evidence to support the proposal that functional alterations in the hippocampus–ventral striatum/pallidum–midbrain pathway relate to the generation of psychotic symptoms.^7^

Data from animal models^7^ suggest that the projections between the ventral striatum/pallidum and the midbrain are GABAergic. In addition, the administration of GABAergic compounds can prevent the emergence of increased dopaminergic activity in animal models of psychosis, and can also normalize hippocampal overactivity and dopamine dysfunction after these have developed.^53^ GABA-A receptor alterations in the striatum of patients with schizophrenia have been found in post-mortem studies^54^ and decreased GABA concentrations in the left basal ganglia have been reported in early-stage schizophrenia.^55^ Moreover, a recent PET study in UHR subjects described a reduced binding potential of GABA-A receptors in the striatum compared with normal controls, and there were trends for this to be inversely correlated with the severity of psychotic symptoms.^56^ Our finding of increased ventral striatum/pallidum to midbrain connectivity is in line with these suggestions that altered GABAergic transmission plays an important role in the development of psychosis.

Animal models of psychosis also predict increased connectivity from the hippocampus to the ventral striatum,^7^ but we did not find evidence that this was greater in UHR subjects than in controls. Because we did not have a large sample of UHR subjects, this may have been due to insufficient statistical power. Another possibility is that alterations in hippocampal–striatal connectivity vary within UHR samples in relation to long-term outcomes. A recent study found that UHR subjects had greater resting regional perfusion in the hippocampus, striatum and midbrain than controls at presentation, but that this only persisted in subjects whose psychotic symptoms had not subsequently improved.^57^ Although the rate of transition to psychosis in our study was consistent with other cohorts,^58^ statistical analysis of long-term outcomes was likely underpowered.

In addition to the above, some further limitations of the current study merit consideration. In part to improve statistical power, we did not separate the ventral striatum from the ventral pallidum in the DCM analysis, and were thus unable to determine whether the enhanced connectivity to the midbrain in UHR subjects involved the ventral striatum or the ventral pallidum. This issue could be addressed in future studies by using larger samples and higher-resolution imaging techniques, which would be of interest given the opposed functions of the ventral striatum and pallidum in the animal model.^7^

In summary, in line with animal and cognitive models of psychosis, our findings suggest that functional alterations in a hippocampal–basal ganglia–midbrain circuit underlie aberrant salience processing and the formation of psychotic symptoms.

## Figures and Tables

**Table 1:** Demographic and clinical characteristics of the study sample

	*Healthy controls (*n=*32)*	*Ultra high risk (*n=*29)*	*Statistics*
Age (mean±s.d.)	23.69±4.08	21.17±3.08	*t*_59_=2.73, *P*=0.008
Gender (F/M)	14/18	16/13	*χ*^2^=0.794, *P*=0.373
Handedness (R/L)	28/4	25/4	*χ*^2^=0.022, *P*=0.881
Years of education (mean±s.d.)	14.06±2.27	12.51±1.92	*t*_59_=2.88, *P*=0.006
Smoker (yes/no)	8/24	13/16	*χ*^2^=2.549, *P*=0.104
Reported previous use of illicit psychoactive substances (yes/no)	19/10	19/13	*χ*^2^=0.621, *P*=0.621
Reported ongoing occasional cannabis use (yes/no)	5/27	5/24	*χ*^2^=0.029, *P*=0.865
Premorbid IQ (NART, mean±s.d.)	110±9.5	114±11.4	*t*_59_=1.241, *P*=0.22
GAF (mean±s.d.)	81.91±10.7	54.57± 6.75	*t*_59_=9.12, *P*=0.0001
CAARMS positive (mean±s.d.)	—	8.07±3.49	—
CAARMS negative (mean±s.d.)	—	7.04±2.82	—
PANSS positive (mean±s.d.)	—	12.8±3.95	—
PANSS negative (mean±s.d.)	—	14.40±4.17	—

Abbreviations: CAARMS, Comprehensive Assessment of AT Risk Mental States; CAARMS positive symptoms were the sum of severity scores for unusual thought content, non-bizarre ideas, perceptual abnormalities and disorganized speed; CAARMS negative symptoms were the sum of severity scores for alogia, avolition/apathy and anhedonia; GAF, global assessment of function; NART, National Adult reading test; PANSS, Positive and Negative Syndrome Scale; s.d., standard deviation.

**Table 2:** Group differences in fMRI task main effects

*fMRI contrast*	*Group comparison*	*Cluster size*	P-*value FWE*_*roi*_	P-*value FWE*_*roi-bc*_	T-*value*	*Z -value*	*Peak MNI coordinates (*x, y, z*)*	*Brain region*
Reward	UHR>HC	57	0.015	0.045	3.96	3.71	−21, −13, −8	L Ventral Pallidum
		11	0.034	0.12	3.67	3.46	18, −7, −5	R Ventral Pallidum
		10	0.013	0.039	3.44	3.27	−15, −16, −11	L Midbrain
		24	0.081	0.243	3.48	3.30	−21, −31, −5	L Hippocampus (subic)
	UHR<HC	NIL						
Novelty	UHR>HC	NIL						
	UHR<HC	NIL						
Aversion	UHR>HC	NIL						
	UHR<HC	NIL						

Abbreviations: FWE_roi_, family-wise error corrected within individual regions of interest; FWE_roi-bc_, family-wise error corrected within Bonferroni-corrected regions of interest; HC, healthy controls; MNI, Montreal Neurological Institute; UHR, ultra high risk.
