# Correction: Altered activation and connectivity in a hippocampal–basal ganglia–midbrain circuit during salience processing in subjects at ultra high risk for psychosis

**DOI:** 10.1038/s41398-018-0189-4

**Published:** 2018-08-31

**Authors:** T. Winton-Brown, A. Schmidt, J. P. Roiser, O. D. Howes, A. Egerton, P. Fusar-Poli, N. Bunzeck, A. A. Grace, E. Duzel, S. Kapur, P. McGuire

**Affiliations:** 1 Department of Psychosis Studies, Institute of Psychiatry, Psychology and Neuroscience King’s College London, London, UK; 2 Monash Alfred Psychiatry Research Centre, Monash University, Melbourne, VIC Australia; 3 Institute of Cognitive Neuroscience, University College London, London, UK; 4 Psychiatric Imaging, MRC Clinical Sciences Centre, Hammersmith Hospital, London, UK; 5 Early Psychosis: Intervention and Clinical-prediction (EPIC) Lab, Department of Psychosis Studies, Institute of Psychology, Psychiatry and Neuroscience, King’s College London, London, UK; 6 OASIS Service, South London and Maudsley (SLaM) NHS Foundation Trusts, Beckenham, UK; 7 Institute of Psychology, University of Luebeck, Luebeck, Germany; 8 Department of Systems Neuroscience, University Medical Center Hamburg-Eppendorf, Hamburg, Germany; 9 Department of Neuroscience, University of Pittsburgh, Pittsburgh, PA USA; 10 Faculty of Medicine, Dentistry and Health Sciences, University of Melbourne, Melbourne, VIC Australia

Correction to: *Translational Psychiatry*; 10.1038/tp.2017.174; published online 3 October 2017.

This Article was originally published under a CC BY-NC-ND 4.0 license, but has now been made available under a CC BY 4.0 license. The PDF and HTML versions of the Article have been modified accordingly.

