# Supplementary material for: Altered activation and connectivity in a hippocampal–basal ganglia–midbrain circuit during salience processing in subjects at ultra high risk for psychosis
Source: Transl Psychiatry. 2017 Oct 3;7(10):e1245–. doi: 10.1038/tp.2017.174 (PMC5682600; doi:10.1038/tp.2017.174)
Supplement: Supplementary Methods [file tp2017174x1.docx]

**Supplementary methods**

**Recognition Task**

At one hour and 24 hours following the conclusion of the online task participants were shown 140 of the picture cues (balanced across all 8 categories) randomly mixed with 68 new distractor pictures (17 indoor-neutral, 17outdoor-neutral, 17 indoor-emotional, 17 outdoor-emotional). The average IAPS emotional rating of the new emotional distractor pictures was matched to the ratings of the studied pictures (supplementary figure 2). Participants received identical verbal and visual instructions for the recognition task, which consisted of 3 judgments cued by text underneath each picture. Participants were asked to indicate with a button press first if they recognised the pictures (‘Old’ or ‘New’) and if they did then rate their confidence in their memory (Duzel et al., 1997). For pictures judged as ‘New’ they were asked to specify their confidence in that judgment (‘Sure’ or ‘Guess’). Finally they were asked to rate how distressing they found the picture when first viewed by moving a slider along a Visual Analogue Scale from ‘Not Distressing’ to ‘Very Distressing’ (figure 2.4). This was to ensure validity of individual experiences of emotional arousal for each emotional category picture. The time limits for the 3 steps in the recognition task were 3000ms, 4000ms and 5000ms respectively.

Analyses of recognition rates utilized measures of both hit rate and discrimination accuracy, correcting for false alarms (‘new’ pictures recognised falsely as old) according to the following formulae from Signal Detection Theory (Corwin, 1994):

- *Hit Rate (HR) = (Hits +0.5)/(Total Old +1)*
- *False Alarm Rate (FAR) = (False Alarms + 0.5)/(Total New + 1)*
- *Discrimination Accuracy (DA) = HR - FAR*

These were then entered into repeated measures ANOVAs in SPSS with Recall session, Reward, Novelty and Emotion as within participant factors, and group as a between participant factor.

**fMRI image acquisition and preprocessing**

MR images were acquired on a 3T Siemens Tim Trio (Siemens Healthcare, Erlangen, Germany) using a 32-channel phased array head coil. Anatomical reference images were acquired using a dual-contrast B1-homogeneity correcting modification of the Magnetization Prepared Rapid Acquisition of Gradient Echo sequence (MP2RAGE) (1). 1mm isotropic resolution was acquired using TR=5s, TE=2.96ms, 256x240x176mm FOV, a 4 degree flip angle, inversion times of 700 and 2500ms, and a parallel imaging factor of 3 in 8m:22s. T2*-weighted echo-planar (EPI) images were acquired for the functional task using 3.5x3.5x3mm resolution in a 225mm in-plane FOV, TR=2s, TE= 31 ms, 80 degree flip angle, 36 slices in each TR, and a parallel imaging factor of 2. These volumes covered the hippocampus, amygdala, brainstem (including diencephalon, mesencephalon, pons, and medulla oblongata), and neocortex excluding the vertex. 788 volumes were acquired per session.

The fMRI data were preprocessed and statistically analyzed using a general linear model (GLM) approach as implemented in SPM8 (http://www.fil.ion.ucl.ac.uk/spm/software/spm8/). Prior to preprocessing, all scans with scan-to-scan movement greater than 1mm translation or 1.5^0^ rotation were automatically detected and checked for artifact, and all scans were further manually checked for other artefacts. Artefactual scans were removed and replaced by time-averaging adjacent scans, and a ‘dummy’ regressor of no interest was created for each participant which corresponded to the removed artefactual scans ensuring these scans were regressed out of analysis. All functional images were processed according to standard procedures in SPM8: realigned to the first volume, normalized by coregistering, segmenting and warping the T1 scan to Tissue Probability Maps (TPMs) in SPM8 and then applying the T1 warping parameters to the functional images, resampled to 2 x 2 x 2 mm, smoothed with an isotropic 8 mm full-width half-maximum Gaussian kernel, highpass-filtered (cutoff 128 s) and globally scaled over voxels and scans. At the first level there were 18 contrasts applied: a statistical model for each participant was computed by applying a canonical Hemodynamic Response Function (HRF (2)) to each of the 8 regressors of interest generated by the SIT corresponding to the time point of picture cue presentation (2[Reward] x 2 [Novelty] x 2[Aversion]). To capture residual task related brain activity we also included convolved regressors corresponding to No-Go cues and at outcomes and an individual participant error covariate corresponding to trials where participants failed to press the button to ‘Go’ trials (Omission Errors), or pressed the button to ‘NoGo’ trials (Commission Errors). Six non-convolved movement-related covariates per session were included (the three rigid-body translations and three rotations determined from initial realignment) and the dummy regressor for removed artefact scans.

**Bayesian model selection (BMS)**

The BMS method rests on comparing the (log) evidence of a predefined set of models (see model space). The model evidence is the probability of observing the empirical data, given a model, and represents a principled measure of model quality derived from probability theory (12). Concretely, it represents the average predicted data under random sampling from the model’s priors or, alternatively, the difference between the accuracy (fit) of a model and its complexity. We used a random-effects BMS approach for group studies, which is capable of quantifying the degree of heterogeneity in a population while being extremely robust to detect potential outliers (13). One common way to summarize the results of random-effects BMS is to report the exceedance probability (EP) of each model (i.e. the probability that this model is more likely than any other of the models tested, given the group data).

**Supplementary Results**

*Recognition discrimination accuracy*

Discrimination accuracy, calculated as hits – false alarms

**Supplementary Figure 1.** fMRI task.

Picture cues varied along 3 dimensions: Reward prediction (indoor-outdoor setting), Novelty (non-prefamiliarised scenes) and Aversion (negative IAPS scenes). Following presentation of each cue (1.5s) participants pressed a button, followed by a fixation cross (1.5-2.5s) and then either a money reward or no reward outcome (0.5s). To ensure sustained attention, 2 ‘NoGo’ trials were inserted where participants were instructed to withhold the button press.

**Supplementary Figure 2.** Recognition task.

3000ms	4000ms	5000ms

Recognition procedure performed at 1hr and 24hr. Participants judged whether scenes were new or old, then rated their confidence in that judgement and how emotionally distressing they found the pictures when first seen.

**Supplementary Figure 3.** fMRI region of interest masks.

Images display an average T1 for healthy control participants with masks superimposed. **(A)** hippocampus and parahippocampal gyrus, **(B)** midbrain and **(C)** ventral striatum/pallidum.

**Supplementary Figure 4.** Model space construction.

6 different variations of DCMs were created depending on where the modulation of rewarding cues exerted its effect (red arrows) on the endogenous connections (black arrows). According to Grace and colleagues (14-16), we first created a base model including reward-induced modulation of hippocampal-striatal-midbrain connections (*model 1*). Building up on this base model, *model 2* is also considering that midbrain dopamine neurons that innervate the VS do also affect the hippocampus input to the VS via potentiation during reward processing (15). Furthermore, given that midbrain dopamine neurons do also directly project to the hippocampus (17) and thereby regulate the entry of information into long-term memory (14), we incorporated this connection into an additional model and allowed reward to modulate it (*model 3*). Although the VS has no direct projection to the hippocampus, a previous fMRI analysis showed effective connectivity from the VS and to the hippocampus during novelty processing (18). In order to consider this VS modulation of hippocampal activity, we additionally added to model 3 a VS-induced non-linear modulation of midbrain to hippocampus connectivity (*model 4*). Finally, based on the fact that midbrain dopamine neurons that innervate the hippocampus are not the same neurons that innervate the VS (19), we include this pathway as endogenous connection into two additional models but did not let reward modulate it (*model 5 and 6*).

**Supplementary figure 5A.** Reaction times to main effects

**Supplementary figure 5B.** Reaction time interactions of Aversion with Reward and novelty

**Supplementary Figure 6 – Recognition task performance**

A. **Hit Rate**

**B. Discrimination Accuracy**

B. **False Alarms**

Mean (SD) A) recognition hit rate (HR), B) discrimination accuracy (DA) and C) false alarms (FA) for the main effects of Reward, Novelty and Aversive (emotional) cues, at 1hr and at 24hr in ARMS and HC groups. Hit rate refers to % old cues correctly recognised as old, false alarms refers to % new cues falsely recognised as old, discrimination accuracy refers to hit rate adjusted for false alarm rate. ^p<0.01 * p<0.05 **p<0.01 ***p<0.001

**Supplementary Figure 7.** Bayesian model selection comparison of family of models in healthy controls and ultra high risk subjects.

Results are expressed in terms of exceedance probability (EP), the relative probability that this model is more likely than any other of the models tested, given the group data. The family of bilinear models was clearly superior relative to the family of non-linear models in healthy controls (EP(F1)=95%, EP(F2)=5%) and ultra high-risk subjects (EP(F1)=98%, EP(F2)=2%)

**Supplementary Figure 7.** Bayesian model selection comparison of single models in healthy controls and ultra high risk subjects.

Results are expressed in terms of exceedance probability (EP), the relative probability that this model is more likely than any other of the models tested, given the group data. BMS comparison of single models revealed that model 1 was the best fitting model in both HCs (EP=81%) and UHR subjects (EP=65%).

**Supplementary Table 1** – task effects in healthy controls in **A)** regions of interest, **B)** whole brain

|  |  | cluster | peak | peak | peak | peak | MNI | | |  |
| --- | --- | --- | --- | --- | --- | --- | --- | --- | --- | --- |
|  | contrast | Equiv k | p(FWE-ROI) | T | equivZ | p(unc) | x | y | z | Location |
| A. Regions of Interest | Reward | 10 | 0.101 | 4.04 | 3.59 | <0.001 | 9 | -28 | -17 | R Midbrain |
|  |  | 1 | 0.69 | 2.88 | 2.69 | 0.004 | 3 | -13 | -11 | R Midbrain |
|  | Novelty | 56 | 0.036 | 4.48 | 3.9 | <0.001 | 27 | -37 | -11 | R PHG/ Hippocampus (subic) |
|  |  |  | 0.062 | 4.23 | 3.73 | <0.001 | 24 | -28 | -17 | R PHG/ Hippocampus (subic) |
|  |  |  | 0.705 | 2.81 | 2.63 | 0.004 | 24 | -34 | -2 | R Hippocampus (subic) |
|  |  | 42 | 0.083 | 4.1 | 3.63 | <0.001 | -18 | -25 | -20 | L PHG/ Hippocampus (subic) |
|  |  |  | 0.089 | 4.07 | 3.61 | <0.001 | -27 | -31 | -17 | L PHG |
|  |  |  | 0.096 | 4.03 | 3.59 | <0.001 | -24 | -37 | -14 | L PHG |
|  |  | 3 | 0.413 | 3.25 | 2.99 | 0.001 | 27 | 5 | -26 | R PHG/entorhinal Cx |
|  |  | 2 | 0.505 | 3.11 | 2.88 | 0.002 | 27 | -1 | -23 | R Amygdala |
|  | Aversion | 191 | <0.001 | 6.84 | 5.3 | <0.001 | -18 | -31 | -2 | L Hippocampus (subic) |
|  |  | 26 | <0.001 | 6.59 | 5.17 | <0.001 | 18 | -34 | 1 | R Hippocampus (CA) |
|  |  | 6 | 0.085 | 4.15 | 3.67 | <0.001 | 39 | -34 | -17 | Right PHG/Fusiform Gyrus |
|  |  | 3 | 0.141 | 3.91 | 3.5 | <0.001 | 27 | 5 | -26 | R Hippocampus (EC) |
|  |  | 5 | 0.553 | 3.11 | 2.88 | 0.002 | -6 | -25 | -11 | L midbrain (SN) |
|  |  |  |  |  |  |  |  |  |  |  |
|  |  |  |  |  |  |  |  |  |  |  |
| B. Whole Brain | Reward | 534 | 0.001 | 7.18 | 5.47 | <0.001 | -39 | -85 | -8 | L occipital cortex |
|  |  |  | 0.001 | 7.08 | 5.42 | <0.001 | -30 | -88 | 13 | L occipital cortex |
|  |  |  | 0.323 | 4.44 | 3.88 | <0.001 | -39 | -64 | -8 | L occipital cortex |
|  |  | 501 | 0.011 | 6 | 4.85 | <0.001 | 36 | -85 | 10 | R occipital cortex |
|  |  |  | 0.148 | 4.84 | 4.15 | <0.001 | 42 | -79 | -5 | R occipital cortex |
|  |  |  | 0.198 | 4.7 | 4.05 | <0.001 | 42 | -64 | -5 | R occipital cortex |
|  |  | 30 | 0.354 | 4.39 | 3.84 | <0.001 | 6 | -28 | -17 | R Midbrain |
|  |  | 80 | 0.786 | 3.8 | 3.41 | <0.001 | -30 | 20 | -8 | L orbitofrontal cortex |
|  |  |  | 0.98 | 3.31 | 3.04 | 0.001 | -33 | 32 | -5 | L orbitofrontal cortex |
|  |  |  | 0.991 | 3.21 | 2.96 | 0.002 | -24 | 29 | -14 | L orbitofrontal cortex |
|  |  | 90 | 0.909 | 3.57 | 3.24 | 0.001 | -24 | -70 | 34 | L occipital cortex |
|  |  | 34 | 0.991 | 3.21 | 2.96 | 0.002 | 45 | 29 | -2 | R orbitofrontal cortex |
|  |  |  | 0.992 | 3.19 | 2.94 | 0.002 | 51 | 29 | 10 | R orbitofrontal cortex |
|  |  |  | 0.999 | 2.99 | 2.78 | 0.003 | 36 | 29 | -2 | R orbitofrontal cortex |
|  | Novelty | 257 | 0.173 | 4.73 | 4.07 | <0.001 | 24 | -40 | -14 | R PHG |
|  |  |  | 0.435 | 4.23 | 3.73 | <0.001 | 24 | -28 | -17 | R PHG |
|  |  |  | 0.507 | 4.13 | 3.66 | <0.001 | 42 | -64 | -5 | R Inferior Temporal Gyrus |
|  |  | 310 | 0.236 | 4.57 | 3.97 | <0.001 | -24 | -40 | -14 | L PHG |
|  |  |  | 0.407 | 4.27 | 3.76 | <0.001 | -27 | -70 | -8 | L PHG |
|  |  |  | 0.531 | 4.1 | 3.63 | <0.001 | -18 | -25 | -20 | L Hippocampus (subic) |
|  |  | 104 | 0.359 | 4.34 | 3.81 | <0.001 | 39 | -85 | 10 | R Occipital Gyrus |
|  |  |  | 0.777 | 3.77 | 3.39 | <0.001 | 33 | -88 | 19 | R Occipital Gyrus |
|  |  | 23 | 0.812 | 3.71 | 3.35 | <0.001 | 27 | 11 | -26 | R Entorhinal Gyrus |
|  |  |  | 0.958 | 3.38 | 3.09 | 0.001 | 27 | 2 | -23 | R Entorhinal Gyrus |
|  |  |  | 0.999 | 2.89 | 2.7 | 0.003 | 36 | 2 | -20 | R Entorhinal Gyrus |
|  | Aversion | 117 | <0.001 | 6.85 | 5.3 | <0.001 | 21 | -7 | -14 | R Amygdala (BL) |
|  |  |  | 0.187 | 3.77 | 3.4 | <0.001 | 18 | -7 | -5 | R Amygdala |
|  |  |  | 0.304 | 3.51 | 3.2 | 0.001 | 12 | -7 | -5 | R Amygdala |
|  |  | 5751 | <0.001 | 12.1 | 7.3 | <0.001 | 45 | -76 | -5 | R Occipital cortex |
|  |  |  | <0.001 | 10.97 | 6.96 | <0.001 | -39 | -46 | -20 | R Fusiform gyrus |
|  |  |  | <0.001 | 10.91 | 6.94 | <0.001 | 42 | -52 | -17 | L Fusiform gyrus |
|  |  | 1296 | <0.001 | 7.43 | 5.59 | <0.001 | -39 | 29 | -14 | L PHG |
|  |  |  | 0.003 | 6.57 | 5.16 | <0.001 | -48 | 20 | 19 | L orbitofrontal cortex |
|  |  |  | 0.018 | 5.82 | 4.75 | <0.001 | -48 | 26 | 7 | L orbitofrontal cortex |
|  |  | 950 | <0.001 | 7.39 | 5.57 | <0.001 | 39 | 11 | 25 | R orbitofrontal cortex |
|  |  |  | 0.012 | 6 | 4.85 | <0.001 | 51 | 29 | 13 | R orbitofrontal cortex |
|  |  |  | 0.022 | 5.73 | 4.69 | <0.001 | 51 | 17 | 28 | R orbitofrontal cortex |
|  |  | 486 | 0.003 | 6.63 | 5.19 | <0.001 | -6 | 50 | 28 | L ventromedial cortex |
|  |  |  | 0.212 | 4.71 | 4.06 | <0.001 | -9 | 29 | 46 | L ventromedial cortex |
|  |  | 68 | 0.191 | 4.76 | 4.09 | <0.001 | 51 | -7 | -17 | R middle temporal gyrus |
|  |  | 37 | 0.195 | 4.75 | 4.09 | <0.001 | -3 | 41 | -17 | L ventromedial cortex |
|  |  |  | 0.52 | 4.2 | 3.71 | <0.001 | -3 | 50 | -14 | L ventromedial cortex |

**Supplementary Table 2** – task effects in UHR participants in **A)** regions of interest, **B)** whole brain

|  |  | cluster | peak | peak | peak | peak | MNI (mm) | | | peak |
| --- | --- | --- | --- | --- | --- | --- | --- | --- | --- | --- |
|  |  | size | p(FWE-corr) | T | equivZ | p(unc) | x | y | z | location |
| Regions of Interest | Reward | 14 | 0.15 | 3.56 | 3.19 | 0.001 | -12 | -13 | -11 | L Internal Globus Pallidus |
|  |  |  | 0.396 | 2.97 | 2.74 | 0.003 | -18 | -7 | -11 | L Amygdala (LB) |
|  |  | 13 | 0.196 | 3.41 | 3.08 | 0.001 | 6 | -22 | -17 | R Midbrain (SN) |
|  |  | 5 | 0.226 | 3.33 | 3.02 | 0.001 | -18 | -31 | -2 | L Hippocampus |
|  |  | 16 | 0.272 | 3.22 | 2.93 | 0.002 | 27 | -31 | -2 | R Hippocampus |
|  |  | 4 | 0.388 | 2.98 | 2.75 | 0.003 | 15 | -7 | -5 | R Internal Globus Pallidus |
|  | Novelty |  |  |  |  |  |  |  |  |  |
|  | Aversion | 200 | 0.001 | 6.32 | 4.91 | <0.001 | -21 | -7 | -23 | L Hippocampus (Subic)/Amygdala (LB) |
|  |  |  | 0.001 | 6.23 | 4.86 | <0.001 | -30 | -10 | -29 | L Fusiform Gyrus/Hippocampus (subic) |
|  |  |  | 0.01 | 5.13 | 4.25 | <0.001 | -30 | -28 | -20 | L PHG (Hippocampus (CA) |
|  |  |  | 0.027 | 4.69 | 3.97 | <0.001 | -30 | -19 | -23 | L PHG/Hippocampus (CA) |
|  |  |  | 0.044 | 4.47 | 3.83 | <0.001 | -18 | -34 | 1 | L Hippocampus (CA) |
|  |  | 11 | 0.054 | 4.37 | 3.77 | <0.001 | 18 | -34 | 1 | R Hippocampus (CA) |
|  |  |  | 0.198 | 3.72 | 3.31 | <0.001 | 21 | -31 | -2 | R Hippocampus (CA) |
|  |  | 86 | 0.074 | 4.22 | 3.67 | <0.001 | 33 | -34 | -17 | R Fusiform Gyrus/Hippocampus (subic) |
|  |  |  | 0.152 | 3.86 | 3.41 | <0.001 | 30 | -19 | -14 | R Hippocampus (CA) |
|  |  |  | 0.16 | 3.83 | 3.39 | <0.001 | 36 | -25 | -20 | R Hippocampus (CA) |
|  |  |  | 0.214 | 3.68 | 3.28 | 0.001 | 27 | -13 | -14 | R Hippocampus (CA) |
|  |  |  |  |  |  |  |  |  |  |  |
| Whole Brain | Reward | 460 | 0.01 | 5.72 | 4.59 | <0.001 | 42 | -70 | -11 | R Inferior Occipital Gyrus |
|  |  |  | 0.019 | 5.4 | 4.41 | <0.001 | 36 | -82 | -11 | R Inferior Occipital Gyrus |
|  |  |  | 0.058 | 4.88 | 4.1 | <0.001 | 30 | -82 | 10 | R Middle Occipital Gyrus |
|  |  | 254 | 0.012 | 5.63 | 4.54 | <0.001 | -36 | -79 | -17 | L Fusiform Gyrus |
|  |  |  | 0.372 | 3.88 | 3.43 | <0.001 | -27 | -61 | -8 | L Fusiform Gyrus |
|  |  |  | 0.379 | 3.87 | 3.42 | <0.001 | -27 | -85 | 4 | L Middle Occipital Gyrus |
|  |  | 21 | 0.382 | 3.86 | 3.41 | <0.001 | 48 | 38 | 7 | R Inferior Frontal Gyrus p. Triangularis |
|  |  | 118 | 0.571 | 3.56 | 3.2 | 0.001 | 0 | -34 | 34 | L Middle Cingulate Cortex |
|  |  |  | 0.702 | 3.37 | 3.05 | 0.001 | -3 | -46 | 19 | L Posterior Cingulate Cortex |
|  |  |  | 0.858 | 3.09 | 2.83 | 0.002 | -12 | -52 | 16 | L Precuneus |
|  |  | 71 | 0.577 | 3.56 | 3.19 | 0.001 | -12 | -13 | -11 | L Internal Globus Pallidus |
|  |  |  | 0.67 | 3.42 | 3.09 | 0.001 | -18 | -28 | -5 | L Hippocampus (Subic) |
|  |  | 29 | 0.675 | 3.41 | 3.08 | 0.001 | 6 | -22 | -17 | R Midbrain |
|  |  |  | 0.829 | 3.15 | 2.88 | 0.002 | 9 | -31 | -11 | R Midbrain |
|  |  | 7 | 0.69 | 3.39 | 3.06 | 0.001 | -45 | 38 | 7 | L Inferior Frontal Gyrus p. Triangularis |
|  |  | 19 | 0.704 | 3.36 | 3.05 | 0.001 | -3 | 26 | -8 | L Anterior Cingulate Cortex |
|  |  | 32 | 0.754 | 3.28 | 2.99 | 0.001 | 18 | -10 | -8 | R Internal Globus Pallidus |
|  |  |  | 0.827 | 3.15 | 2.88 | 0.002 | 6 | -7 | -11 | R Midbrain |
|  |  | 21 | 0.793 | 3.22 | 2.93 | 0.002 | 27 | -31 | -2 | R Hippocampus (CA) |
|  |  | 22 | 0.875 |  | 2.8 | 0.003 | -39 | 23 | -2 | L Inferior Frontal Gyrus p. Triangularis |
|  |  | 4 | 0.919 | 2.93 | 2.71 | 0.003 | 30 | 2 | -23 | R Amygdala (LB) |
|  | Novelty | 181 | 0.117 | 4.78 | 4.03 | <0.001 | -27 | -58 | -14 | L Fusiform Gyrus |
|  |  |  | 0.251 | 4.37 | 3.77 | <0.001 | -39 | -67 | -8 | L Inferior Occipital Gyrus |
|  |  | 84 | 0.326 | 4.22 | 3.67 | <0.001 | 48 | -67 | -5 | R Inferior Temporal Gyrus |
|  |  |  | 0.843 | 3.41 | 3.08 | 0.001 | 42 | -52 | -8 | R Inferior Temporal Gyrus |
|  |  |  | 0.993 | 2.81 | 2.61 | 0.005 | 36 | -70 | -8 | R Inferior Occipital Gyrus |
|  |  | 26 | 0.502 | 3.93 | 3.47 | <0.001 | 33 | -79 | 13 | R Middle Occipital Gyrus |
|  |  | 10 | 0.961 | 3.09 | 2.83 | 0.002 | -15 | -34 | 22 | L Cingulate Gyrus |
|  |  | 5 | 0.984 | 2.93 | 2.71 | 0.003 | -42 | 8 | 31 | L Precentral Gyrus |
|  | Aversion | 2260 | <0.001 | 10.44 | 6.55 | <0.001 | -39 | -55 | -17 | L Fusiform Gyrus/PHG |
|  |  |  | <0.001 | 10.33 | 6.52 | <0.001 | -39 | -67 | -14 | L Fusiform Gyrus/PHG |
|  |  |  | <0.001 | 8.76 | 5.98 | <0.001 | -42 | -64 | 16 | L Middle Temporal Gyrus |
|  |  | 1584 | <0.001 | 9.61 | 6.28 | <0.001 | 42 | -76 | -2 | R Inferior Occipital Gyrus |
|  |  |  | <0.001 | 8.96 | 6.05 | <0.001 | 39 | -49 | -17 | R Fusiform Gyrus |
|  |  |  | <0.001 | 8.72 | 5.96 | <0.001 | 42 | -61 | -11 | R Inferior Temporal Gyrus |
|  |  | 102 | 0.001 | 7.1 | 5.29 | <0.001 | 0 | 50 | -17 | L Rectal Gyrus |
|  |  | 543 | 0.019 | 5.79 | 4.63 | <0.001 | 39 | 8 | 31 | R Inferior Frontal Gyrus p. Opercularis |
|  |  |  | 0.051 | 5.33 | 4.37 | <0.001 | 39 | 35 | -14 | R Inferior Frontal Gyrus p. Orbitalis |
|  |  |  | 0.066 | 5.21 | 4.29 | <0.001 | 48 | 32 | 10 | R Inferior Frontal Gyrus p. Triangularis |
|  |  | 249 | 0.029 | 5.6 | 4.52 | <0.001 | 0 | 56 | 22 | L Superior Medial Gyrus |
|  |  |  | 0.969 | 3.23 | 2.94 | 0.002 | -15 | 38 | 37 | L Superior Frontal Gyrus |
|  |  | 48 | 0.117 | 4.93 | 4.13 | <0.001 | 15 | -31 | 1 | R Thalamus |
|  |  | 178 | 0.124 | 4.9 | 4.11 | <0.001 | -3 | -55 | 19 | L Precuneus |
|  |  | 32 | 0.169 | 4.74 | 4.01 | <0.001 | -39 | 17 | -35 | L Medial Temporal Pole |
|  |  |  | 0.454 | 4.16 | 3.63 | <0.001 | -45 | 8 | -35 | L Inferior Temporal Gyrus |
|  |  | 104 | 0.209 | 4.63 | 3.94 | <0.001 | 57 | -4 | -17 | R Middle Temporal Gyrus |
|  |  |  | 0.404 | 4.24 | 3.68 | <0.001 | 48 | -13 | -14 | R Middle Temporal Gyrus |
|  |  |  | 0.617 | 3.93 | 3.46 | <0.001 | 42 | 17 | -32 | R Medial Temporal Pole |
|  |  | 21 | 0.486 | 4.12 | 3.59 | <0.001 | 24 | -52 | 43 | L SPL |
|  |  | 39 | 0.52 | 4.07 | 3.56 | <0.001 | -54 | -7 | -17 | L Middle Temporal Gyrus |
|  |  | 4 | 0.812 | 3.64 | 3.25 | 0.001 | 39 | -13 | -29 | R Hippocampus (CA) |

**Supplementary Table 3. Dynamic Causal Modelling Parameter Estimates**

|  | **Healthy controls (n=32)** | **High-risk subjects (n=28)** |
| --- | --- | --- |
| **hippocampus->ventral striatum** | 0.052073 ± 0.025181^*^ | 0.050406 ± 0.027978 |
| **ventral striatum/pallidum->midbrain^+^** | 0.018339 ± 0.006530^*^ | 0.063128 ± 0.013439^*^ |
| **midbrain->hippocampus** | 0.005350 ± 0.001212^*^ | 0.013664 ± 0.004812^*^ |
| **midbrain->ventral striatum** | 0.045862 ± 0.009149^*^ | 0.023408 ± 0.019672 |

Mean ± SE for the modulatory effect induced by reward-indicating cues (posterior means obtained from Bayesian model averaging).

* Significant t tests within each group compared with zero (p< 0.05).

^+^ Significant between-group effect (t=-3.117, p = 0.003).

**References**

1. Marques JP, Kober T, Krueger G, van der Zwaag W, Van de Moortele PF, Gruetter R (2010): MP2RAGE, a self bias-field corrected sequence for improved segmentation and T1-mapping at high field. *Neuroimage*. 49:1271-1281.
2. Friston KJ, Josephs O, Rees G, Turner R (1998): Nonlinear event-related responses in fMRI. *Magn Reson Med*. 39:41-52.
3. Egerton A, Chaddock CA, Winton-Brown TT, Bloomfield MA, Bhattacharyya S, Allen P, et al. (2013): Presynaptic striatal dopamine dysfunction in people at ultra-high risk for psychosis: findings in a second cohort. *Biol Psychiatry*. 74:106-112.
4. Turkheimer FE, Aston JA, Asselin MC, Hinz R (2006): Multi-resolution Bayesian regression in PET dynamic studies using wavelets. *Neuroimage*. 32:111-121.
5. Studholme C, Hill DL, Hawkes DJ (1996): Automated 3-D registration of MR and CT images of the head. *Med Image Anal*. 1:163-175.
6. Martinez D, Slifstein M, Broft A, et al. Imaging human mesolimbic dopamine transmission with positron emission tomography. Part II: amphetamine-induced dopamine release in the functional subdivisions of the striatum. *J Cereb Blood Flow Metab*. 2003;23(3):285-300.
7. Hammers A, Allom R, Koepp MJ, Free SL, Myers R, Lemieux L, et al. (2003): Three-dimensional maximum probability atlas of the human brain, with particular reference to the temporal lobe. *Hum Brain Mapp*. 19:224-247.
8. Hartvig P, Agren H, Reibring L, et al. Brain kinetics of L-[beta-11C]dopa in humans studied by positron emission tomography. *J Neural Transm Gen Sect*. 1991;86(1):25-41.
9. Hoshi H, Kuwabara H, Léger G, Cumming P, Guttman M, Gjedde A. 6-[18F]fluoro-L-dopa metabolism in living human brain: a comparison of six analytical methods. *J Cereb Blood Flow Metab*. 1993;13(1):57-69. doi:10.1038/jcbfm.1993.8.
10. Patlak CS, Blasberg RG. Graphical evaluation of blood-to-brain transfer constants from multiple-time uptake data. Generalizations. *J Cereb Blood Flow Metab*. 1985;5(4):584-590. doi:10.1038/jcbfm.1985.87.
11. Egerton A, Demjaha A, McGuire P, Mehta MA, Howes OD (2010): The test-retest reliability of 18F-DOPA PET in assessing striatal and extrastriatal presynaptic dopaminergic function. *Neuroimage*. 50:524-531.
12. Penny WD, Stephan KE, Mechelli A, Friston KJ (2004): Comparing dynamic causal models. *Neuroimage*. 22:1157-1172.
13. Stephan KE, Penny WD, Daunizeau J, Moran RJ, Friston KJ (2009): Bayesian model selection for group studies. *Neuroimage*. 46:1004-1017.
14. Lisman JE, Grace AA (2005): The hippocampal-VTA loop: controlling the entry of information into long-term memory. *Neuron*. 46:703-713.
15. Sesack SR, Grace AA (2010): Cortico-Basal Ganglia reward network: microcircuitry. *Neuropsychopharmacology*. 35:27-47.
16. Lodge DJ, Grace AA (2011): Developmental pathology, dopamine, stress and schizophrenia. *Int J Dev Neurosci*. 29:207-213.
17. Gasbarri A, Sulli A, Packard MG (1997): The dopaminergic mesencephalic projections to the hippocampal formation in the rat. *Prog Neuropsychopharmacol Biol Psychiatry*. 21:1-22.
18. Kafkas A, Montaldi D (2015): Striatal and midbrain connectivity with the hippocampus selectively boosts memory for contextual novelty. *Hippocampus*.
19. Swanson LW (1982): The projections of the ventral tegmental area and adjacent regions: a combined fluorescent retrograde tracer and immunofluorescence study in the rat. *Brain Res Bull*. 9:321-353.
